# Surgical explantation of an infected Lotus Edge valve: a case report

**DOI:** 10.1186/s44215-024-00178-y

**Published:** 2024-12-20

**Authors:** Yusuke Yanagino, Satoshi Kainuma, Naonori Kawamoto, Naoki Tadokoro, Takashi Kakuta, Ayumi Ikuta, Kohei Tonai, Tomoyuki Fujita, Satsuki Fukushima

**Affiliations:** Department of Cardiovascular Surgery, National Cerebral and Cardiovascular Center, Osaka, 564-8565 Japan

**Keywords:** Lotus Edge valve, Transcatheter aortic valve replacement, Transcatheter aortic valve replacement valve explantation, Prosthetic valve endocarditis, Annular abscess

## Abstract

**Background:**

With the rapid expansion of transcatheter aortic valve replacement (TAVR), TAVR valve explantation is also increasing. Nevertheless, previous reports on Lotus Edge valve explantation are limited to only two reports, none of which include intraoperative videos. Therefore, we report the case of an older adult who underwent a 2-year-old Lotus Edge valve explantation, after developing prosthetic valve endocarditis (PVE) and aortic annular abscess, with a strong indication for a TAVR explantation and surgical aortic valve replacement (AVR).

**Case presentation:**

An 85-year-old male patient, who underwent TAVR with a 25-mm Lotus Edge valve for severe aortic stenosis 2 years ago, was referred to our hospital. He presented with a 1-month history of high-grade fever, refractory to oral antimicrobials and trifascicular heart block. Two sets of blood cultures were positive for *Streptococcus dysgalactiae* subspecies *equisimilis*, and transesophageal echocardiography revealed vegetation on the valve leaflets. Enhanced computed tomography scan showed thickening and enhancement of the aortic root and aorto-mitral continuity, with a small low-density area. Therefore, we diagnosed PVE. Subsequently, we planned AVR re-intervention and pacemaker implantation. The vegetation mass was attached to the aortic valve leaflet. We attempted to explant the valve while deforming it using forceps. The areas with abscess formation were easily dissected; however, the other areas were difficult to separate. Cold-saline irrigation softened the nitinol stent and enabled to dissect the prosthetic valve from the aortic wall. The infected aortic annulus was irrigated and then repaired. AVR using a 21-mm Avalus bioprosthetic valve and epicardial pacemaker lead implantation were simultaneously performed. Postoperative echocardiography confirmed that the prosthetic valve function was favorable, and the patient was transferred to a rehabilitation hospital after 6 weeks of intravenous antimicrobial therapy.

**Conclusion:**

The Lotus Edge valve is difficult to remove due to its fixation after deployment and strong adhesion, but the use of cold water may be effective in facilitating its removal.

**Supplementary Information:**

The online version contains supplementary material available at 10.1186/s44215-024-00178-y.

## Background

The Lotus Edge valve (Boston Scientific, Natick, MA, USA) was the third transcatheter aortic valve replacement (TAVR) valve approved in Japan, following SAPIEN (Edwards Lifesciences, Irvine, CA, USA) and CoreValve (Medtronic, Minneapolis, MN, USA). This valve can reduce paravalvular aortic regurgitation using “adaptive seal” system. The valve begins to function early in the delivery, hemodynamics are stable, and ventricular pacing is unnecessary [1]. However, due to concerns about the delivery system, it was withdrawn from the market in 2021 [2].

With the rapid expansion of TAVR to low-risk and younger patients, TAVR explantation is also increasing [3]. Nevertheless, Lotus Edge valve explantations are limited to two reports, without intraoperative videos [4, 5]. Consequently, herein, we present the case of an older adult who underwent a 2-year-old Lotus Edge valve explantation, after developing prosthetic valve endocarditis (PVE) and aortic annular abscess, with a strong indication for a TAVR explantation and surgical aortic valve replacement (AVR).

## Case presentation

An 85-year-old male patient, who had a TAVR with a 25-mm Lotus Edge valve for severe aortic stenosis 2 years ago, was referred to our hospital. He presented with 1-month history of fever over 39 °C or higher, refractory to oral antimicrobials and trifascicular heart block. Transesophageal echocardiography revealed vegetation on the valve leaflets and thickened tissue across the aorto-mitral continuity, reaching the mitral annulus. Contrast-enhanced computed tomography scan also revealed thickening and enhancement of the aortic root and aorto-mitral continuity, with a small low-density area observed. Two sets of blood cultures were positive for *Streptococcus dysgalactiae* subspecies *equisimilis*; thus, we diagnosed PVE with aortic annular abscess. Despite the patient’s Society of Thoracic Surgeons score of 17.1%, we scheduled a TAVR explantation, AVR re-intervention, and pacemaker implantation.

**Fig. 1:**
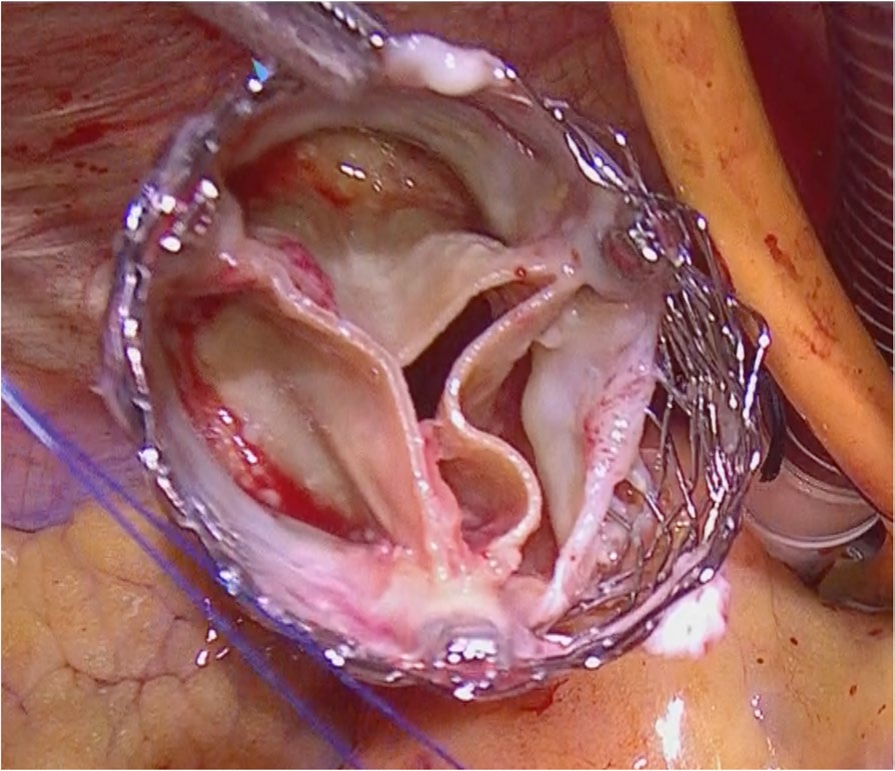
Infected prosthetic valve

Surgery was performed through a median sternotomy, utilizing standard cardiopulmonary bypass support and cardioplegic arrest. The vegetation mass was attached to the aortic valve leaflet (Fig. 1). We attempted to explant the valve while deforming it using forceps. However, since the Lotus valve becomes fixed after deployment, deformation was extremely difficult. Additionally, the proximal portion of the prosthetic valve was severely adhered to the endothelium, at the level of the valve skirt. While the areas with abscess formation were easily dissected, the other areas were difficult to separate. Cold-saline irrigation softened the nitinol stent, facilitating prudent, circumferential dissection of the prosthetic valve from the native tissue. After explant the Lotus valve, we found the annular abscesses were observed in two locations: from the left coronary cusp to the noncoronary cusp and from the noncoronary cusp to the right coronary cusp. The abscess at the noncoronary cusp was extensive, with some areas extending to the subaortic curtain. We carefully excised the native valve and debrided the damaged annular tissue. Fortunately, the mitral valve and its surrounding tissues appeared to be preserved. However, due to the loss of annular tissue in the areas where the abscesses had formed, we reconstructed it using two rectangular bovine pericardial patches. AVR using a 21-mm Avalus bioprosthetic valve (Medtronic, Minneapolis, MN, USA) and epicardial pacemaker lead implantation were simultaneously performed (see Additional file 1). Postoperative echocardiography revealed a preserved left ventricular ejection fraction, mean trans-aortic pressure gradient, and effective orifice area (EOA) of 50%, 10 mm Hg, and 1.39 cm^2^ (EOA index was 0.88), respectively, with mild paravalvular aortic regurgitation (PVL). Six-week post-antimicrobial therapy, he was discharged to a rehabilitation hospital.

The removed prosthetic valve has a vegetation mass adhering to the entire valve. Bacterial culture tests revealed the presence of the same microbes as those present in the blood culture

## Discussion and conclusions

We report the surgical explantation of Lotus valve 2 years after implantation due to PVE. There are many reports of surgical removal of TAVR valves, but most of them are related to SAPIEN or Evolute valves [3, 6, 7]; only a few reports of surgical removal of Lotus valves exist [4, 5]. Although the Lotus valve is no longer available, after confirming with Boston Scientific, we learned that there have been 92 Lotus valve implantations in Japan, including those conducted in clinical trials. Internationally, there have been more than 700 implantations [8, 9, 10], based on documented cases alone. This report may provide important insight into the procedure to remove the Lotus Edge valve.

There are two reports detailing useful techniques to ensure safe removal of TAVR valve: the “double Kocher clamp technique [3]” and a procedure in which the valve is cut longitudinally and retracted with a Kelly klamp [11]. In the case of the Lotus valve, due to its unique structure that is fixed after final deployment, we considered that the previously reported methods could not be directly applied. We inquired with a proctor from Boston Scientific in the USA about the valve explantation method, but we were informed that there is no standardized procedure for removal, and that cutting the valve skirt should be avoided due to concerns about sudden deformation of the valve. We attempted to deform the prosthetic valve using forceps; however, the valve was too hard to fold sufficiently to remove. Therefore, we applied cold water to soften the woven nitinol used in the frame of the Lotus valve and succeeded in achieving sufficient folding of the valve to remove it. [5, 12]

Since the aortic valve annulus was destroyed due to infection, we thoroughly debrided and reconstructed with bovine pericardial patches, and an Avalus 21 mm was implanted. Aortic root replacement was also considered, but since the patient was elderly and the surgical risk was very high [6, 7], early completion of the operation was the priority. We did consider the use of a sutureless valve. However, although there is one reported case of using a sutureless valve for an annular abscess [13], such reports are extremely limited. So, we chose to secure the valve with sutures in the standard manner to ensure a more reliable fixation. Postoperatively, the patient had residual mild PVL, but no severe patient prosthesis mismatch with an EOA index of 0.88 and a low mean pressure gradient of 10 mmHg, and no new mitral regurgitation was observed. If the operative risk is very high, partial aortic annulus repair may be effective to reduce aortic cross-clamp time.

Thus, this report of an uncommon case of Lotus Edge valve explantation will better equip surgeons in the safe execution thereof.

## Supplementary Information

Additional file 1: Surgical explant of Lotus valve. The infected prosthetic valve is removed with softening by cold-water irrigation. This technique allowed removal of the valve in its entire circumference without damaging the native annulus. The annular abscess was thoroughly debrided and reconstructed using a bovine pericardial patch, followed by aortic valve replacement.

## Data Availability

Data sharing is not applicable to this article as no datasets were generated or analyzed during the current study.

## Abbreviations

TAVR: Transcatheter aortic valve replacement
AVR: Aortic valve replacement
PVE: Prosthetic valve endocarditis
EOA: Effective orifice area
PVL: Paravalvular aortic regurgitation
